# Maternal Depression and Its Association With Child Undernutrition in Urban Ethiopia

**DOI:** 10.1155/ijpe/7491512

**Published:** 2026-06-16

**Authors:** Sitota Gebremelak, Tefera Darge Delbiso

**Affiliations:** ^1^ School of Public Health, Addis Ababa University, Addis Ababa, Ethiopia, aau.edu.et

**Keywords:** anxiety, depression, mental disorder, PHQ–9, undernutrition

## Abstract

Maternal depression is one of several multidimensional factors that influence children′s nutritional status. However, research in this area remains limited in urban poverty settings, where residents are often assumed to have better access to socioeconomic and health services. We estimated the prevalence of maternal depression and assessed its association with undernutrition among children aged 12–36 months of economically disadvantaged mothers in urban Ethiopia. A cross‐sectional survey was conducted to collect data from 627 mother‐child pairs in selected urban areas of Ethiopia: Adama, Addis Ababa, Debre Birhan. Maternal depression was measured using Patient Health Questionnaire–9 (PHQ–9) score, whereas child nutritional status was measured using anthropometric methods. Background characteristics of the study were summarized using percentages and averages. Binary logistic regression was used to assess the association between maternal depression and undernutrition, adjusting for confounders. The prevalence of maternal depression was 16.0% (95% CI: 13.3%–19.0%), while stunting among children aged 12–36 months was 52.7% (95% CI: 48.7%–56.7%). Maternal depression was significantly associated with child stunting after adjusting for covariates but showed no significant association with wasting or underweight. Children of mothers exhibiting depression symptoms had 2.21 times higher odds of being stunted (95% CI: 1.33–3.68) compared with those of nondepressed mothers. These findings underscore the critical need for integrated public health interventions that incorporate maternal mental health into child nutrition programs, alongside strengthened community advocacy and education on maternal mental health. Childcare support with income‐generating activities for women can boost women′s income, provide greater peace of mind, and support improved mental well‐being.

## 1. Introduction

Mental disorders have become a critical public health concern and are now among the leading contributors to the global burden of disease [1, 2]. Its prevalence continues to rise, driven largely by depression and anxiety. The largest increases in mental disorder were observed following the COVID‐19 pandemic, although the prevalence had already been rising steadily over the past decades [3]. Mental disorders are particularly prevalent among women and young age group [1], as well as among uneducated, widowed, separated, or divorced women and those from poorer households [4]. In 2020, depression disorders affected approximately 3153 people per 100,000 population worldwide, equivalent to 246 million individuals. In the same year, the prevalence in Sub‐Saharan Africa was 2988 per 100,000 population, representing roughly 35 million of the population [1]. Despite their health risks and contribution to mortality, anxiety and depression alone cost the global economy more than $1 trillion annually [5].

In low‐ and middle‐income countries (LMICs), the prevalence of maternal depression ranges from 15.6% during pregnancy to 19.8% during postpartum period. In Sub‐Saharan Africa, 6%–30% of women experience maternal depression during the postpartum period, and about 20% of suicidal attempts occur during this time [5]. Maternal depression tends to be more prevalent in urban areas, often due to living without partner, financial constraint, and postpartum health complications. Maternal depression is among the major public health concerns in Ethiopia but largely neglected. An estimated 12.2%–33.8% mothers suffered from depression during postpartum period, however, an estimated 76%–85% of depressed mothers are neither diagnosed nor treated [6].

Although the global prevalence of child stunting declined from 33% to 22% between 2000 and 2022, it remains an alarming public health challenge in many developing countries, particularly in Africa [7], where it contributes to half of all deaths among children under five. Despite Ethiopia has been implementing a range of nutrition programs and interventions over the past three decades, including the National Nutrition Program I and II, the National Nutrition‐Sensitive Agriculture, School Feeding Programs, the Productive Safety Net Programs, and various micronutrient supplementation initiatives, the prevalence of stunting remains very high at 40% [4, 8]. The country is off track of achieving most of the Sustainable Development Goals (SGDs), particularly SDG 2.2, end all forms of malnutrition by 2030 [9], and the Seqota declaration goal to end stunting in children under two by 2030 [10].

Several complex, multidimensional, and interrelated factors influence the nutritional status of child, maternal depression is considered as one of them [11, 12]. Healthy maternal behavior and attitude have an essential role in maintaining healthy nutrition in children. Maternal depression is associated with poor child growth outcomes, limited parent‐child interaction, and delayed child development. These effects are largely due to reduced mother′s responsiveness to care for herself and her baby [13, 14], which can lead to undernutrition. Undernutrition, in turn, impairs cognitive development, lowers school performance, increases the likelihood of school dropout, and decreases future productivity [11, 15]. However, some studies have found no association between maternal depression and stunting among children [16, 17]. Despite substantial evidence linking maternal depression to adverse child nutrition outcomes [18–22]), research on this area remains largely overlooked in urban poverty settings, where residents are often assumed to have better access to socioeconomic and health services. This gap limits a detailed understanding of how maternal depression contributes to childhood undernutrition and hinders the development of context‐specific interventions. Therefore, this study is aimed at estimating the prevalence of maternal depression and assess its association with undernutrition among children aged 12–36 months of economically disadvantaged mothers in urban Ethiopia.

## 2. Materials and Methods

### 2.1. Study Design and Areas

This study used data from the baseline household survey conducted as part of the growth and economic opportunity for women (GrOW)—East Africa project, using a community‐based cross‐sectional study design. The project is aimed at assessing whether subsidized and enhanced quality community‐based childcare services can improve women′s well‐being and empowerment in Ethiopia. The project was implemented by Child Fund Ethiopia, Child Believe Fund, and Tesfa Berhan Child and Family Development. The survey was carried out in urban areas: Addis Ababa (the capital city of Ethiopia), Debre Berhan (a city in the Amhara region and located about 120 km northeast of Addis Ababa), and Adama (a city in the central Oromia region and located about 100 km southeast of Addis Ababa) between July and September 2022. Details of the study can be found in (Study ID: RIDIE‐STUDY‐ID‐63bf977db6192).

### 2.2. Study Population, Sample Size, and Sampling Procedure

All women who were unemployed or had irregular jobs, primarily due to childcare responsibilities, and who had children aged 12–36 months living in the selected urban areas (Adama, Addis Ababa, Debre Birhan) were eligible for inclusion in the study. The sample size was determined using a single population proportion formula [23], assuming a 95% confidence level, a 5% margin of error, a design effect of 2, a stunting prevalence of 26% in urban Ethiopia based on [24], and a 5% nonresponse rate. This yielded a sample size of 622, which is slightly less than the number of study participants included in the baseline survey, which was 629 mother‐child pairs. Therefore, this study used the full sample of 629 to increase statistical power.

The baseline survey encompassed 14 woredas/kebeles (the smallest level of government structure in Ethiopia), from seven subcities where implementing partners were operating. Specifically, Lemi Kura subcity was one of the seven subcities selected from Addis Ababa, where two woredas were chosen. In Debre Berhan town, seven kebeles were selected from four subcities, whereas in Adama, five woredas were selected from the Bole and Abba Geda subcities. A sampling frame was compiled, including all women from the selected woredas/kebeles who met the inclusion criteria and consented to participate in the project. From the list, a random sample of 629 women‐child pairs was selected: 154 each from Addis Ababa and Adama, and 324 from Debre Birhan. These participants took part in the baseline.

### 2.3. Variables and Measurements

#### 2.3.1. Outcome Variable

The outcome variable of this study is stunting among children aged 12–36 months. The length or height of each child was measured to the nearest 0.1 cm using a portable hardwood board with a movable headpiece. Measurements were taken in duplicate and checked for discrepancies. If the difference exceeded the acceptable limit of 1.0 cm, the measurements were repeated twice, and then the mean value was recorded. The anthropometric index, height‐for‐age z‐score (HAZ), weight‐for‐height z‐score (WHZ), and weight‐for‐age z‐score (WAZ) were calculated using the WHO Child Growth Standard with WHO Anthro 3.2.2 software. Children with HAZ, WHZ, or WAZ values < –2 standard deviations (SD) from the median on the WHO Child Growth Standards were classified as stunted, wasted, or underweight, respectively, whereas those with values ≥ –2SD were categorized as having normal nutritional status [25].

#### 2.3.2. Exposure Variables

Maternal depression was the exposure variable and measured using Patient Health Questionnaire–9 (PHQ–9), an instrument widely used to identify symptoms of depression. The tool consists of nine occurrence questions, each followed by three frequency‐of‐occurrence questions. These items assess the individual′s mood, sleep pattern, appetite, energy levels, thoughts, and behavior over the past 2 weeks. The PHQ–9 score ranges from 0 to 27, with each of the nine items scored from 0 (not at all) to 3 (nearly every day), where a higher total score indicates severe depression [26]. For the descriptive analysis, the PHQ–9 score was categorized according to increasing depression severity: no or minimal depression (0–9), mild depression [10–14], and moderate to severe depression [15–27]. A PHQ–9 score of 10 or higher indicates the presence of depression and is used for the multivariable analysis. This tool has been validated in Ethiopia, both in urban and rural areas, with a sensitivity of 86% and a specificity of 92% in urban settings [27].

#### 2.3.3. Covariates

Several covariates were considered in this study, selected based on literature reviews and their availability in surveyed data. These covariates include child‐related (age, sex, and illness history in the past 2 weeks), mothers‐related (age, marital status, education level, and maternal nutritional status measured using body mass index [BMI]), and household‐related (household food security, wealth index, source of drinking water, and type of toilet facility).

The wealth index, a composite measure of household living standards, was calculated using principal component analysis based on asset ownership, materials used to construct the house, drinking water and sanitation facilities, and household income [28]. Households were then categorized into three: poor, medium, and rich. Household food insecurity status was assessed using the Household Food Insecurity Access Scale (HFIAS), which includes nine occurrence questions (yes or no) and corresponding frequency questions to capture experiences of food access over the past 4 weeks. The questions cover three areas: anxiety about food supply, insufficient food quality, and inadequate food intake. Based on the total HFIAS score, households were classified into four categories: food secure, mildly, moderately, and severely food insecure [29].

### 2.4. Data Collection and Quality Assurance

Standard and validated questionnaires were developed in English and translated into Amharic language (widely used local language). The questionnaire was pretested before the actual data collection and finally was designed into a digital data collection platform, KoboCollect, to enhance data quality and efficiency. Qualified and experienced data collectors were used. The survey team was given a 3‐day training followed by a pilot survey in Addis Ababa to familiarize themselves with the tool. During data collection, supervisors reviewed each questionnaire for completeness and consistency before submission, whereas the research team monitored submissions throughout the survey period.

### 2.5. Data Analysis

Prior to analysis, the data was cleaned and coded to ensure quality. The anthropometric index, HAZ, WHZ, and WAZ were calculated using WHO Anthro software. Following the WHO flagging convention, biologically implausible values were identified as HAZ < −6 or > +6 SD (10 children; 1.6% of the total), WHZ < −5 or > +5 SD (12 children; 1.9% of the total), and WAZ < −6 or > +5 SD (2 children; 0.3% of the total), relative to the median z‐score of the WHO Child Growth standards [25]. These values were excluded from the analysis. The background characteristics of the study units were described and summarized using percentage, and median (interquartile ranges [IQR]). A bivariate analysis was performed to screen potential covariates for inclusion in the multivariable model. Covariates associated with undernutrition at a significant level of *p* value < 0.25 were considered eligible for inclusion in the multivariable analysis. Then, a binary logistic regression was conducted to assess the association between maternal depression and undernutrition, adjusting for the eligible covariates. Model fit was evaluated using the Hosmer–Lemeshow test, which indicated a good fit (*p* value = 0.441). Multicollinearity was evaluated using variance inflation factors (VIF), with all VIF values < 3.0, suggesting the absence of significant collinearity among predictors. Statistical significance was confirmed at a *p* value < 0.05. Adjusted odds ratio (OR) with 95% confidence interval (CI) were estimated and reported to measure the strength of the association. The data analysis was conducted using SPSS Version 27.0.1.

### 2.6. Ethical Consideration

Ethical clearance was obtained from the Addis Ababa University College of Health Sciences Institutional Review Board (IRB; 053/22/SPH). All the study participants were informed about their voluntary participation and their liberty to withdraw from the research at any time without explanation and/or prejudice. Then, voluntary verbal informed consent was obtained from the mothers, and parental/guardian assent was obtained for the children.

## 3. Results and Discussion

### 3.1. Results

#### 3.1.1. Characteristics of Children, Mothers, and Households

Of the total 629 mother‐child pairs surveyed, 627 were included in this analysis, resulting in a response rate of 99.7%. The median age of children was 26 months, with 56.6% aged between 24 and 36 months, and 52.5% were boys. Two in five children reported an illness in the 2 weeks preceding the survey. The median age of mothers was 28 years. The majority of the women were followers of the Orthodox religion (84.7%), one‐fourth were either divorced, separated, or widowed, and 18.3% had never attained school. The prevalence of overweight was 22.5% among the women. More than one‐fourth (27.8%) of the women were heads of their households. About 37% of households had a family size of more than four, 97% had access to improved drinking water, 62% had improved toilet facilities, and 96.1% experienced either mild, moderate, or severe food insecurity (Table 1).

**Table 1:** Percentage distribution of the socioeconomic and demographic characteristics of study participants in selected urban Ethiopia, July–September 2022 (*N* = 627).

Characteristics	*n* (%)
**Child characteristics**	
Sex (boys)	329 (52.5)
Age in months, median (IQR)	26 (20–32)
Age group	
12–23 months	272 (43.4)
24–36 months	355 (56.6)
Child was ill 2 weeks preceding the survey	254 (40.5)
**Maternal characteristics**	
Age in years, median (IQR)	28 (25–33)
Education	
No education	115 (18.3)
Primary	277 (44.2)
Secondary or higher	235 (37.5)
Marital status	
Married	433 (69.1)
Single	39 (6.2)
Divorced or separated or widowed	155 (24.7)
Religion	
Orthodox	531 (84.7)
Muslim	71 (11.3)
Protestant and others	25 (4.0)
Maternal nutritional status (BMI)	
Normal	421 (67.1)
Underweight	65 (10.4)
Overweight	141 (22.5)
Women headed household	174 (27.8)
**Household characteristics**	
Household size (> 4 members)	229 (36.5)
Household food security status	
Food secure	31 (4.9)
Food insecure	596 (96.1)
Source of drinking water	
Improved	608 (97.0)
Unimproved	19 (3.0)
Type of toilet facility	
Improved	389 (62.0)
Unimproved	238 (38.0)
Wealth index	
Rich	209 (33.3)
Middle	213 (34.0)
Poor	205 (32.7)

#### 3.1.2. Prevalence of Maternal Depression and Undernutrition

The prevalence of maternal depression (defined as mothers exhibiting moderate to severe depressive symptoms based on the PHQ–9) was 16.0% (**95% CI: 13.3%–19.0%), whereas mild depression accounted for 41.3% (95% CI: 37.5%–45.2%), and no or minimal depression for 42.7% (95% CI: 38.9%–46.7%). The prevalence of moderate to severe depression varied significantly across the study areas: the** highest rate was observed in Addis Ababa at 27.0% (95% CI: 20.5%–34.6%), followed by Debre Berhan at 17.2% (13.5%–21.7%), and Adama at 2.0% (95% CI: 0.6%–6.0%).

**Figure 1:**
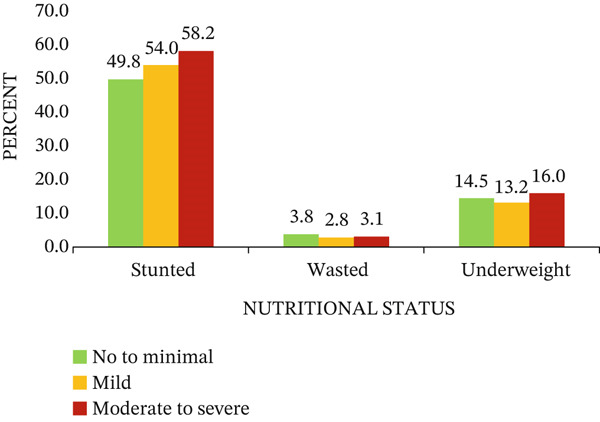
Depression severity and nutritional status in selected urban areas of Ethiopia, July–September 2022.

The prevalence of stunting among children aged 12–36 months was 52.7% (95% CI: 48.7%–56.7%), with 27.4% (95% CI: 24.0%–31.0%) classified as severely stunted. The prevalence of underweight was 14.0% (95% CI: 11.3%–16.7%), while wasting accounted for 3.6% (95% CI: 2.1%–5.0%). Although the association is not statistically significant, maternal depression is related to child nutritional status, with moderately or severely depressed mothers more likely to have undernourished children (Figure 1).

#### 3.1.3. Association Between Maternal Depression and Undernutrition

Maternal depression was significantly associated with stunting after adjusting for covariates. The odds of stunting were 2.21 times higher (95% CI: 1.33–3.68) among children born from mothers with moderate to severe depression compared with those born to nondepressed mothers. Additionally, child sex, mothers′ educational level, family size, episode of diarrhea, and food insecurity were significantly associated with child stunting. The odds of stunting were higher among male children than female children (AOR = 1.5). Increased odds were also observed among children of mothers with no education (AOR = 1.98) or primary education (AOR = 1.59) compared with those whose mothers had secondary or higher education; among children from households with more than four members (AOR = 1.77); among children who experienced diarrhea in the 2 weeks preceding the survey (AOR = 3.79); and among children from food‐insecure households compared with food‐secure households (AOR = 2.80) (Table 2).

**Table 2:** Association between maternal depression and stunting among children aged 12–36 months in selected urban areas of Ethiopia, adjusted for socioeconomic and health‐related covariates.

Characteristics	Stunted, *n* (%)	Not‐stunted, *n* (%)	COR (95% CI)	AOR (95% CI)	*p*
Maternal depression					
Depressed	71 (72.4)	27 (27.6)	2.73 (1.70, 4.40)	2.21 (1.33, 3.68)	0.001
Not depressed	256 (49.0)	266 (51.0)	1.0	1.0	
Child sex					
Male	189 (58.2)	136 (41.8)	1.58 (1.15, 2.17)	1.49 (1.06, 2.11)	0.023
Female	138 (46.8)	157 (53.2)	1.0	1.0	
Child age					
12–23 months	161 (60.5)	105 (39.5)	1.0	1.0	
24–36 months	166 (46.9)	188 (53.1)	0.58 (0.42, 0.80)	0.57 (0.39, 0.79)	0.001
Education					
No education	72 (63.2)	42 (36.8)	2.11 (1.33, 3.34)	1.98 (1.21, 3.25)	0.007
Primary	150 (55.1)	122 (44.9)	1.15 (1.06, 2.15)	1.59 (1.08, 2.32)	0.018
Secondary or higher	105 (44.9)	129 (55.1)	1.0	1.0	
Household headship status					
Male	224 (49.8)	226 (50.2)	0.65 (0.45, 0.92)	0.71 (0.48, 1.05)	0.087
Female	103 (60.6)	67 (39.4)	1.0	1.0	
Household size					
≤ 4	187 (47.3)	208 (52.7)	1.0	1.0	
> 4	140 (62.2)	85 (37.8)	1.83 (1.31, 2.56)	1.77 (1.22, 2.55)	0.002
Diarrhea status					
Yes	253 (48.2)	272 (51.8)	3.79 (2.67, 6.33)	3.79 (2.67, 6.33)	0.001
No	74 (77.9)	21 (22.1)	1.0	1.0	
Household food security status					
Food secure	9 (29.0)	22 (71.0)	1.0	1.0	
Food insecure	318 (54.0)	271 (46.0)	2.87 (1.30, 6.34)	2.80 (1.17, 6.69)	0.021
Type of toilet facility					
Improved	189 (49.2)	195 (50.8)	1.0	1.0	
Unimproved	138 (58.2)	98 (41.5)	1.45 (1.05, 2.02)	1.31 (0.92, 1.88)	0.141

*Note:* *n* = sample size; 1 = reference category.

Abbreviations: AOR, adjusted odd ratio; COR, crude odds ratio.

However, maternal depression was not significantly associated with child underweight (OR = 1.12; 95% CI: 0.50–2.10; *p* = 0.762) after adjusting for covariates. On the other hand, maternal education, maternal nutritional status, family size, and household wealth were significant associations with underweight. Children of mothers with primary level education had higher odds of underweight than those with secondary or higher education (AOR = 1.83). The odds were also higher among children of underweight mothers (AOR = 2.59), in households with more than four members (AOR = 2.21), and in poor (AOR = 3.55) and medium‐wealth households (AOR = 2.27) compared with those from rich families (Table 3).

**Table 3:** Association between maternal depression and underweight among children aged 12–36 months in selected urban areas of Ethiopia, adjusted for socioeconomic and health‐related covariates.

Characteristics	Underweight, *n* (%)	Normal weight, *n* (%)	COR (95% CI)	AOR (95% CI)	*p*
Maternal depression					
Depressed	15 (85.0)	85 (85.0)	1.10 (0.60–2.00)	1.12 (0.50–2.10)	0.762
Not depressed	73 (13.9)	454 (86.1)	1	1	
Education					
No education	11 (9.6)	104 (90.4)	0.82 (0.39–1.71)	0.62 (0.28–1.35)	0.231
Primary	50 (18.1)	227 (81.9)	1.70 (1.03–2.81)	1.83 (1.08–3.10)	0.026
Secondary or higher	27 (11.5)	208 (88.5)	1	1	
Maternal nutritional status					
Normal	59 (14.0)	363 (86.0)	1	1	
Underweight	17 (26.6)	47 (73.4)	2.23 (1.20–4.13)	2.59 (1.32–5.07)	0.001
Overweight	12 (8.5)	129 (91.5)	0.57 (0.30–1.10)	0.54 (0.28–1.06)	0.074
Family size					
≤ 4	44 (11.1	354 (88.9)	1	1	
> 4	44 (19.2)	185 (80.8)	1.91 (1.22–3.01)	2.21 (1.32–3.69)	0.002
Wealth index					
Poor	44 (21.0)	166 (79.0)	3.60 (1.90–6.80)	3.55 (1.83–5.07)	0.001
Medium	30 (14.1)	183 (85.9)	2.23 (1.14–4.33)	2.27 (1.14–4.51)	0.019
Rich	14 (6.9)	190 (93.1)	1	1	
Toilet facility					
Improved	46 (11.8)	343 (88.2)	1	1	
Unimproved	42 (17.6)	196 (82.4)	1.60 (1.02–2.52)	1.40 (0.86–2.17)	0.172

*Note:* *n* = sample size; 1 = reference category.

Abbreviations: AOR, adjusted odd ratio; COR, crude odds ratio.

Given the low prevalence of wasting (3.6%), multivariable regression analysis was not performed, as the model would have limited power to detect meaningful associations.

### 3.2. Discussion

This study estimated the prevalence of maternal depression and assessed its association with undernutrition among children 12–36 months old in urban Ethiopia. The findings are aimed at generating evidence that can inform context‐specific, integrated interventions addressing both maternal mental health and child nutrition.

The findings indicate that the prevalence of maternal depression was 16.0% (95% CI: 13.3%–19.0%). **This estimate is lower than the 21% prevalence reported in a recent national survey in Ethiopia [** **4** **].** It is important to note, however, that the national estimate was based on the WHO′s Self‐Reporting Questionnaire (SRQ‐20), which may contribute to differences in reported prevalence. Similarly, a study conducted in Gondar specialized hospital, Ethiopia, reported a higher prevalence of 36.4% [19], likely due to its hospital setting, where mothers were exposed to a stressful hospital environment that could increase the likelihood of maternal depression. In contrast, this result is consistent with previous studies conducted in Ghana [30] and Bangladesh [31], where the prevalence of maternal depression was reported as 16.8% and 16.7%, respectively.

Although the prevalence of wasting (3.6%) and underweight (14.0%) in our study was comparable to estimates from the recent national demographic and health survey for urban areas (5% and 15.5%, respectively), the prevalence of stunting (52.7%) was substantially higher than the 31% reported nationally for urban populations [4]. This difference may be explained by characteristics of our study population, particularly the economic disadvantage of mothers, which may have limited their capacity to provide adequate resources for optimal child growth and nutrition. Further details on undernutrition prevalence and its determinants in the study area can be found in [32].

Maternal depression was significantly associated with stunting but not with wasting or being underweight, and these associations persisted after adjusting for socioeconomic and health‐related confounders. The odds of stunting among children from depressed mothers were 2.21 times (95% CI: 1.33–3.68) higher compared with nondepressed mothers. This is mainly because maternal depression contributes to poor feeding practices and weakening mother‐child bonding [18, 33, 34] thereby increasing the likelihood of early cessation of exclusive breastfeeding. It may also reduce the mother′s ability to maintain hygiene practices, which affects child‐eating behaviors and increases their susceptibility to childhood illness [35]. Depressed mothers are also more likely to be unemployed or unable to provide adequate food for their families [16, 18], which can contribute to undernutrition. Additionally, depressive and anxiety disorders can contribute to economic and health shocks [1, 18], which in turn may impair a mother′s ability to care for her children, impact food security, and increase the risk of undernutrition.

This finding is consistent with studies conducted in western and northern Ethiopia, where maternal depression was significantly associated with stunting after adjusting for covariates [20, 34]. Similarly, a study conducted in Uganda [22] showed that maternal depression was associated with a 2.4‐fold increase in the risk of stunting, whereas evidence from Pakistan [21] reported a 3.15‐fold increase. Overall, maternal depression is responsible for 3.2 million excess cases of childhood stunting in developing countries, including 1.1 million in Sub‐Saharan Africa [18].

Interventions involving early screening, diagnosis, and treatment of maternal depression during the first 1000 days are critical in reducing child undernutrition. In addition to its impact on child nutrition outcomes, addressing maternal depression can also influence underlying determinants of malnutrition, such as poverty and food insecurity [18]. In resource‐limited settings such as ours, integrating income‐generation opportunities for women with context‐specific maternal depression treatment may enhance maternal well‐being and improve child nutrition outcomes [18]. In Ghana, for example, specialized social support for depressed mothers contributed to reducing the negative impact of depression on child nutrition [17]. In this regard, providing childcare support can free women′s time to work while ensuring their children are cared for, thereby boosting women′s employment, increasing household income, and ultimately contributing to improved nutrition outcomes. Moreover, increased income can reduce finance‐related anxiety and dependence on others, giving women greater peace of mind and supporting improved mental well‐being.

Our study has some limitations that should be considered during interpretation of the results. First, the cross‐sectional design of the study may limit the ability to explain the causal relationship between maternal depression and child undernutrition. Therefore, we recommend future research using a longitudinal design. Second, the study participants of this research were economically disadvantaged mothers of children aged 12–36 months; therefore, generalizing the result to other groups should be made with caution. Third, the use of a self‐report questionnaire to assess maternal depression may have introduced recall bias and social desirability bias; however, these were mitigated by employing standardized and validating tools, ensuring confidentiality, and utilizing trained enumerators during data collection.

## 4. Conclusion

The study found a significant association between maternal depression and stunting, where children born to depressed mothers had more than twice the odds of being stunted compared with children born to nondepressed mothers. The study recommends integration of maternal mental health services into maternal and child health programs at primary healthcare levels to ensure early screening and support. Scaling up community advocacy and education on maternal mental health through sustained campaigns can strengthen community understanding and support. Integrating childcare support with income‐generating activities for women can boost women′s income, provide greater peace of mind, and support improved mental well‐being.

## Funding

No funding was received for this manuscript.

## Conflicts of Interest

The authors declare no conflicts of interest.

## Data Availability

The data that support the findings of this study are available from the corresponding author upon reasonable request.
